# PhyloPro2.0: a database for the dynamic exploration of phylogenetically conserved proteins and their domain architectures across the Eukarya

**DOI:** 10.1093/database/baw013

**Published:** 2016-03-15

**Authors:** Graham L. Cromar, Anthony Zhao, Xuejian Xiong, Lakshmipuram S. Swapna, Noeleen Loughran, Hongyan Song, John Parkinson

**Affiliations:** 1 Program in Molecular Structure and Function, Hospital for Sick Children, 21-9830 PGCRL, 686 Bay Street, Toronto, ON M5G 0A4, Canada and; 2 Departments of Biochemistry, Computer Science and Molecular Genetics, University of Toronto, Toronto, ON M5S 1A8, Canada

## Abstract

PhyloPro is a database and accompanying web-based application for the construction and exploration of phylogenetic profiles across the Eukarya. In this update article, we present six major new developments in PhyloPro: (i) integration of Pfam-A domain predictions for all proteins; (ii) new summary heatmaps and detailed level views of domain conservation; (iii) an interactive, network-based visualization tool for exploration of domain architectures and their conservation; (iv) ability to browse based on protein functional categories (GOSlim); (v) improvements to the web interface to enhance drill down capability from the heatmap view; and (vi) improved coverage including 164 eukaryotes and 12 reference species. In addition, we provide improved support for downloading data and images in a variety of formats. Among the existing tools available for phylogenetic profiles, PhyloPro provides several innovative domain-based features including a novel domain adjacency visualization tool. These are designed to allow the user to identify and compare proteins with similar domain architectures across species and thus develop hypotheses about the evolution of lineage-specific trajectories.

**Database URL**: http://www.compsysbio.org/phylopro/

## Introduction

Phylogenetic profiling has been widely adopted as a method to visualize evolutionary conservation of genes/proteins. This approach has been facilitated by improvements in sequencing technology resulting in an ever increasing number of fully sequenced, eukaryotic genomes. Aside from using phylogenetic profiles to predict gene function (1–3), a number of online tools have been developed to allow users to explore and visualize phylogenetic profiles. For the most part, such tools are restricted to providing profiles for a single orthologous group of proteins (orthogroup). For example, EnsemblCompara GeneTrees, which is largely focused on vertebrates, allows the visualization of ortholog gains and losses in the context of a phylogenetic tree (4). TreeFam offers a summary tree visual, which indicates the proportion of species within lineages that possess orthologs of a selected gene (5), while EggNOG (6) and OrthoMCL (7) provide the ability to generate a taxonomic profile for a specified orthogroup as well as to identify genes with defined phylogenetic profiles. However these tools do not allow the visualization of more than one orthogroup at a time and in the case of EnsemblCompara and Treefam, are largely focused on vertebrates and metazoa, respectively. On the other hand, the OMA (8) database resource, which captures orthologous relationships from 1706 complete proteomes, does offer the capacity to view taxonomic profiles for closely related orthogroups. However, it does not allow the direct comparison of potentially unrelated orthogroups; furthermore, the lack of clustering of profiles makes it difficult to infer lineage-specific innovations across large groups of genes.

Phylogeny based methods, which are computationally intensive, provide robust support for orthology focused at the level of individual genes. PhylomeDB (9), for instance, provides phylogenetic trees and alignments as well as orthology and parology predictions for a seed sequence based on calling duplication and speciation events on the tree as determined by a species-overlap algorithm. This differs from the more commonly used approach of reconciling a gene tree to the species tree. As a consequence of building gene trees for every gene, multi-gene families are represented by several, independently derived trees. MetaPhOrs (10) has exploited this additional information to define a measure of reliability for ortholog predictions based on consistency. MetaPhOrs applies this approach to trees derived from PhylomeDB (9), EnsemblCompara (4), TreeFam (11) and yeast Orthogroups (12) as well as trees reconstructed from EggNOG (6), OrthoMCL (7) and COG (13) to provide phylogeny-based orthology and paralogy predictions for 4.1 million proteins in 829 fully sequenced genomes. A disadvantage remains that this focused approach comes at the expense of tools to analyze/visualize higher level patterns at the level of functionally related pathways or complexes. Recently, there has been much debate in the orthology community about whether orthology/paralogy predictions which are traditionally genocentric are not more appropriately made at the domain-level or smaller (14). Protein domains are conserved, relatively short units of selection [typically <200 amino acid residues (15)] that mostly correspond to independent folding units. According to this argument, differences in the linear sequence of domains (deemed the domain architecture) can be common among orthologs and are functionally important. This is especially apparent for multicellular eukaryotes where domain architectures are often highly complex and lineage specific (14, 16). For example, within the drosophilids, domain rearrangements were found to occur in 36% of gene families (17). Consequently, while genocentric methods remain a common approach, a re-examination of the assumptions and definitions underlying orthology prediction has resulted in a community consensus that a range of tools will continue to be required, and is indeed desirable, in addressing different orthology-based questions in a variety of contexts (18).

Underscoring their importance, ortholog databases are beginning to include domain level information, for example, the latest release of PhylomeDB (9) includes information on Pfam-A domains. However, aside from the ability to reference and view domain architectures on individual gene trees, there is currently a lack of further facility to explore domain adjacency patterns or overview domain conservation at the level of the phylogeny. The contribution of novel domains and domain combinations in driving protein evolution has been a subject of much recent interest (19–23). In addition to emphasizing the potential for differing mechanisms to contribute to domain variation in different lineages, these studies reveal that patterns of selective domain gain or loss, including the gain or loss of domain repeats, contribute to the evolutionary trajectory of species. To our knowledge, no sequence-based orthology databases yet incorporate domain-level information to help delineate orthology assignments.

Addressing this gap, we present PhyloPro2.0, a database integrating pre-calculated, Inparanoid-based orthologs. Here, we rely on Inparanoid orthology assignments as it is a well-established, BLAST-based method which has been shown to perform as well as or better than other methods across a wide range of eukaryotic genomes with both sensitivity and specificity >80% (24). Due to the reliance of PhyloPro2.0 on pairwise orthology assignments, Inparanoid is well suited to our automated workflow. Unlike existing resources that focus on individual orthogroups, PhyloPro2.0 offers the capacity to visualize and study orthology and domain phyletic profiles for large sets of genes (up to 1000 genes) and their orthologs. Further, through an interactive domain adjacency visualization tool, users are able to explore the influence of domain architectures on protein conservation. These data and tools, based on highly confident Pfam-A domain predictions (25), enable the user to link overall protein conservation with underlying domain conservation patterns. PhyloPro has proved to be a valuable resource for evolutionary and comparative studies of biological systems, such as Chromatin modification (26), Extracellular matrices (27), Apicomplexan membrane proteins (28) and vertebrate multi-domain proteins (23). PhyloPro is freely available via the web and all underlying datasets are downloadable.

## PHYLOPRO2.0: generating evolutionary trajectories

### Features

Since its original publication (29) PhyloPro has been continuously updated. Novel in the current release we include Pfam domain and domain architecture conservation information and tools for their exploration across clades. The heatmap view and clustering capability featured in the protein and domain conservation views are unique to PhyloPro and provide a powerful tool for systems level assessment of broad conservation patterns. Clustering is accomplished using Cluster3.0 (30) and can be customized prior to visualization using the advanced search tool. One of the strengths of PhyloPro is the ability to visualize relationships across many closely related species. This allows the identification of inconsistencies such as the absence of orthologs across a specific lineage that may indicate issues in orthology assignment (potentially due to quality of the associated genome). Compared with the previous release the number of reference organisms has doubled (from 6 to 12) and the number of available genomes has expanded (from 120 to 164).

### Data acquisition

Focusing on 12 model organisms (Table 1), the Inparanoid algorithm (31) was used to perform pairwise homology searches for each model species against 164 eukaryotes (including other model species) for which a complete genome sequence has been generated. These comprise 6 plant species, 3 green algae, 1 red algae, 4 stramenopiles, 1 haptophyte, 2 ciliates, 13 apicomplexans, 4 kinetoplastids, 1 diplomonad, 1 cryptophyte, 1 parabasilid, 1 heterolobosid, 2 amoebazoa, 1 microsporidium, 36 fungi, 5 ‘basal’ metazoa, 8 lophotrochozoa, 16 nematodes, 21 arthropods, 4 chordates, 9 vertebrates and 24 mammals. The use of Inparanoid readily facilitates the identification of so called in-paralogs representing lineage-specific gene duplication events. Given a list of query genes from a model species (the ‘reference’ species), for each ‘target’ species, we define one of five possible homology relationships: (i) no detectable ortholog, (ii) one to one (1:1)—a single query gene has a single ortholog in the target species, (iii) one to many (1:M)—a single query gene has two or more orthologs in the target species, (iv) many to one (M:1)—a query gene together with at least one additional paralog are orthologs of a single gene in the target species, and (v) many to many (M:M)—a query gene together with at least one additional paralog are orthologs of at least two orthologs in the target species genome. The collation of these relationships for each of the 164 target species defines a phylogenetic profile for each query gene which is stored along with the domain predictions described below in a local PostgreSQL database. We also make these datasets, together with the proteome datasets, available for download.

**Table 1..** List of reference species

No.	Common name	Scientific name	Source (Date)
1	Thale cress	*Arabidopsis thaliana*	PlantGDB: v.173 (26/08/09)
2	Bakers yeast	*Saccharomyces cerevisiae*	SGD: (12/12/07)
3	Roundworm	*Caenorhabditis elegans*	WormBase: WS205 (30/07/09)
4	Fruit fly	*Drosophila melanogaster*	FlyBase: v.1.3 (25/06/09)
5	House mouse	*Mus musculus*	ENSEMBL: (23/11/07)
6	Human	*Homo sapiens*	ENSEMBL: (23/11/07)
7	Malarial parasite	*Plasmodium falciparum* 3D7	PlasmoDB: v.5.4 (24/09/07)
8	Toxoplasma parasite	*Toxoplasma gondii* ME49	ToxoDB: v.4.3 (01/11/07)
9	Zebrafish	*Danio rerio*	ENSEMBL: (23/11/07)
10	Fission yeast	*Schizosaccharomyces pombe*	SANGER: (11/05/06)
11	Leishmania parasite	*Leishmania major* strain Friedlin	EMBL: (24/12/07)
12	Blood Fluke	*Schistosoma mansoni*	ENSEMBL: (31/07/14)

Domain predictions, based on Pfam-A definitions, were performed on a parallel computing platform using HMMER 3.0 with default parameters as implemented in PfamScan (32). Data flow was handled in a data processing pipeline written in house using Perl. Note Pfam defines six types of entries: Family, Domain, Repeat, Motifs, Coiled-Coil and Disordered (http://pfam.xfam.org/help). For our analysis, we only included Pfam-A definitions for entries labelled as either ‘Domains’ (defined by Pfam as a ‘structural unit’) or ‘Families’ (defined by Pfam as ‘a collection of related protein regions’), as these best fit our criteria as independent folding units. Domains found in each of the proteins in the 12 reference sequences were compared with domains representing the full proteome of every other species. Domain architectures of target and reference orthologs were compared and classified parsimoniously as having gained or lost domains, having the same (conserved) domain architecture, or having rearrangements. Where more than one sequence of gains, losses or rearrangements were equally parsimonious, this resulted in a classification of ‘complex’ type. For purposes of the comparisons, domain order was taken into account. Adjacent domains were defined in the N-terminal to C-terminal orientation. Reverse orientations were considered to be unique (i.e. A − B ≠ B − A).

Functional annotations (GOSlim) for human proteins were acquired from BioMart (33). We used Ensembl 80 with default parameters and the following additional filters: Status (gene): KNOWN, Status (transcript): KNOWN, Transcript Support Level (TSL): Only, Limit to genes: with Pfscan ID(s). The frequencies of the resulting annotations were calculated using a perl script and available functional categories were limited to a subset with frequencies below what we considered to be a reasonable threshold of 1000 proteins for bulk search.

### Querying and browsing in PhyloPro

PhyloPro features several ways to launch a search. A quick search using default options can be performed by entering a space separated list of gene or protein identifiers for a select reference species of interest into the search box, selecting the type of information to return (protein conservation, domain conservation or domain adjacency) and clicking on the ‘Go’ button. For quick searches, the default reference organism for comparison corresponds to the type of identifier first identified among the first 10 listed genes. For example, the use of a mouse gene identifier (e.g. ENSMUSG00000034205) would result in an analysis with mouse as the reference species. Identifier types are not limited to Ensembl but reflect a variety of identifiers in use for various species, depending on cross-referencing available at the time the species was loaded. Alternatively, users have the option of choosing a functional category from a list of Gene Ontology (GO) terms to automatically populate the search list with proteins annotated to the selected term. For performance considerations, available terms are based on GOSlim annotations with a frequency cutoff of 1000 proteins.

Beyond the quick search and GO browsing capabilities, PhyloPro also offers a search option based on sequence similarities, using the well-established BLAST algorithm. It is recognized that as genomes and gene models become updated, gene and protein identifiers may become obsolete. The inclusion of the sequence similarity search option is introduced to guards against such possibilities. After selecting this option from the home page, the user is presented with a sequence similarity search page with options to run a nucleotide-based (BLASTx) or protein-based (BLASTp) search against a reference proteome of their choice. The user pastes in a set of sequences in fasta format and after clicking the ‘Go’ button, PhyloPro retrieves the top BLAST hit associated with each query sequence. The resulting page (protein conservation, domain conservation or domain network view as selected by the user) is then constructed from these hits. Mappings of the user sequences to the identified hits are also presented.

Finally, PhyloPro also offers an advanced search option that allows users to specify a number of parameters for the analysis including: (i) choice of reference species, (ii) limit the range of target species, (iii) choose the similarity metric and clustering method used for clustering the resulting heatmap (if applicable), (iv) choose the type of view (as above) and (v) upload a text file corresponding to the proteins to be searched. Users can review the selected parameters before clicking ‘Go’ to start the search. It is worth mentioning that there is a slight difference in the search depending on whether the user chooses to use a gene identifier vs. a protein identifier in the search box. The use of gene identifiers will result in PhyloPro finding the longest peptide of those which map to the selected gene identifier as the basis for orthology prediction, whereas the use of a protein identifier will result in a protein conservation profile including the exact protein specified as the reference. By design, all domain-based views use the longest peptide for the corresponding gene as the basis for domain comparisons. PhyloPro uses a PostgreSQL (http://www.postgresql.org/) database to speed the retrieval of large amounts of pre-calculated orthology and domain predictions. After a few moments the user will be taken to one of three views depending on their initial choice.

**Figure 1.**
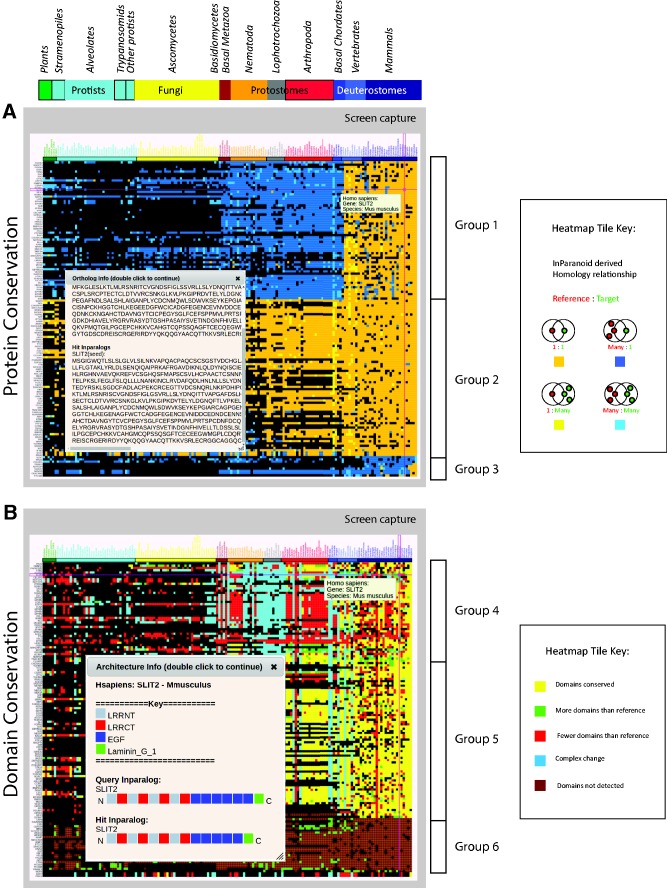
Protein and domain conservation views. (**A**) Conservation of proteins corresponding to the GOSlim category, ‘Anatomical structure formation involved in morphogenesis’. Colored tiles indicate the presence (color) or absence (black) of an ortholog of the reference organism (in this case human) in a given target species. Species are indicated across the top, grouped by phylogeny with plants on the left. Proteins are indicated in rows on the left, clustered so that proteins with similar patterns of conservation are grouped together. The sequence of a selected human reference protein (SLIT2) and its mouse ortholog are also shown (inset). (**B**) Domain architecture conservation corresponding to the same group of proteins as in (A) above. Tile colors reflect the comparison between the reference and target domain architectures. The corresponding architectures for SLIT2 are shown (inset). Note that gene order is determined by clustering and is independent between views.

The protein conservation view (Figure 1A) displays a heatmap in which colored tiles indicate the presence (color) or absence (black) of an ortholog of the reference organism in a given target species. The exact reference protein and target species corresponding to a particular tile is revealed by a mouse-over event, and selecting the tile displays the protein sequence of the orthologs and any predicted inparalogs arising from one to many (1:M), many to one (M:1) or many to many (M:M) predictions (Figure 1A inset). The heatmap presented shows a subset of proteins corresponding to the GO functional category, ‘Anatomical structure formation involved in morphogenesis’ with human as the reference organism. For consistency, we use this subset as the basis for subsequent figures. Clustering of this set revealed at least three potentially interesting groupings corresponding to genes of mostly metazoan origin whose genomes have acquired additional paralogs in vertebrates (Group 1), a group including highly conserved genes with few additional paralogs (Group 2) and a smaller group consisting of some genes of mammalian origin as well as those featuring primate-specific paralogs (Group 3). Amongst the Group 1 proteins is SLIT2, a protein thought to act as a molecular guidance cue in cellular migration (34). Among an assortment of similar functions, SLIT1 and SLIT2 appear to be essential for midline guidance in the forebrain, acting as a repulsive signal preventing inappropriate midline crossing by axons projecting from the olfactory bulb. This may explain the occurrence of additional paralogs in vertebrates. The heatmap image, summary analysis as well as the underlying sequence data may be downloaded from the view.

The domain conservation view (Figure 1B) displays a heatmap similar in layout to the protein view, with black tiles indicating the absence of an ortholog in the target species. However, here tiles are colored to indicate inferences about domain gain, loss or rearrangement resulting from a comparison of domain architecture (defined as the linear sequence of domains in the N to C terminal direction) in the reference vs. target orthologs. For simplicity, gains and losses of domains in repeats are grouped with those for single domains for purposes of coloring tiles in this view. However, a more granular categorization is captured in the summary analysis that can be downloaded along with the image and underlying domain architectures from this view. As with the protein view, a mouse-over event reveals the reference protein and target species corresponding to a particular tile. Selecting the tile displays the domain architecture for the reference and target sequence. Here, we once again focus on the mouse ortholog of the SLIT2 protein which is a red tile indicating fewer domains than the human reference. The pop-up allows us to identify that the mouse ortholog is very similar to the reference protein with a difference of one EGF domain (Figure 1B inset). Clustering by domain conservation has revealed at least three broad categories of possible interest. The first indicates a subset of proteins (Group 4) characterized by a high variability in their domain architectures with possible clade-specific differences in the lophotrochozoa and arthropods, perhaps corresponding to morphological differences in life cycles. The second group (Group 5) appears to have largely conserved domain architectures, whereas the last group (Group 6) consists of proteins with no detectable domains. The latter may occur for reasons such as high sequence divergence or poor sequence quality in which case the domains that may be present remain below the confidence threshold.

### A novel interactive domain visualization tool

**Figure 2.**
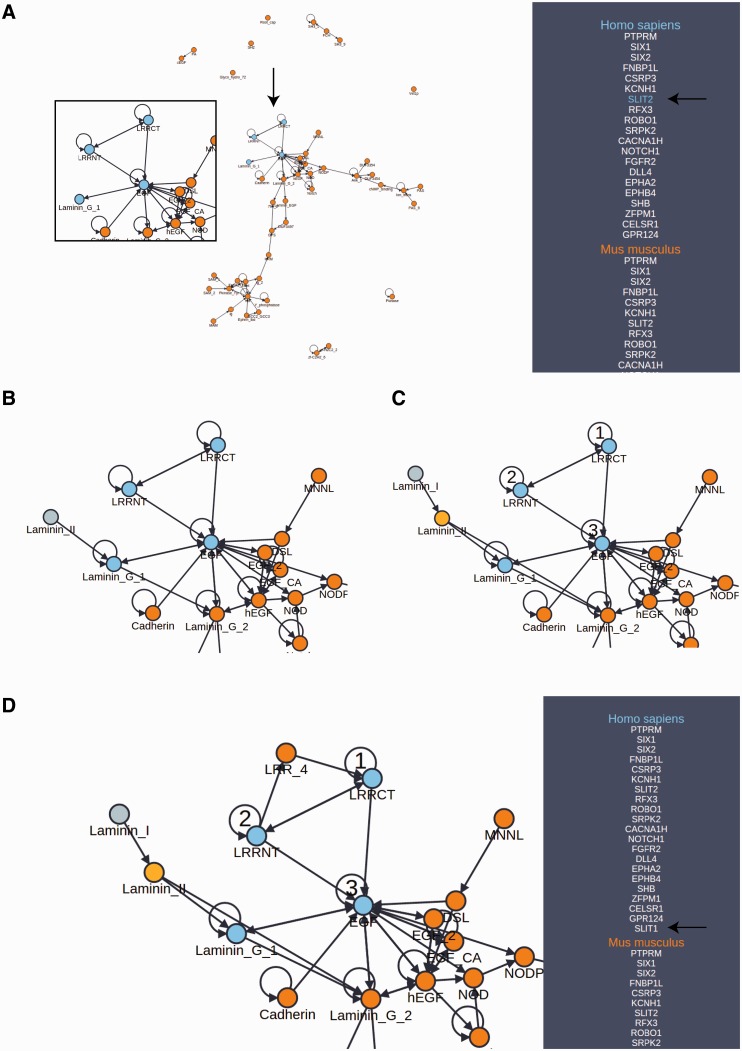
Domain adjacency network exploration. (**A**) A domain adjacency graph for a subset of proteins corresponding to the GOSlim category, ‘Anatomical structure formation involved in morphogenesis’. Domains are shown as nodes. Edges indicate the adjacency of domain pairs (N to C terminal direction) within one or more architectures corresponding to the searched proteins listed in the side panel. For the example protein (SLIT2), the highlighted nodes indicate the domain architecture pertaining to this protein (enlargement and arrows added for emphasis). The side panel lists the orthologs of the searched proteins from which the graph has been constructed. (**B**) The area of interest has been expanded from the Laminin_G_1 node to include an additional Laminin_II domain, indicating that this duo appears in one or more additional proteins not in the original search. (**C**) Expansion continues with Laminin_II now added to the network as a permanent addition, further expansion from this domain identifies Laminin_I as a new neighbor. Selection of numbered nodes, presents a green ‘Protein Search’ button which initiates a search for additional proteins with this architecture that are not in the original list of search proteins. (**D**) The search in (C) has returned one additional protein (SLIT1) which was not in the original list of searched proteins. Exploration from LRRCT reveals LRR_4 as an adjacent neighbor. Note that multiple adjacent domains are often returned from the search allowing one to build up a rich network in the direction of interest. Also, by selecting the ortholog in another species, differences in architectures between species may be explored and expansions may be scoped to a particular species.

Domain architectures may be further explored using the domain adjacency view (Figure 2). Here, selected proteins for the reference organism are listed on the right panel along with the names of orthologous proteins among 12 model species comprising the set of possible reference species. In the provided example, we selected the set of all reference species as the basis for this view because they represent an informative cross section of the available phylogeny. The main view consists of a directed network connecting domains (nodes) into architectures (a linear sequence of domains connected by edges in the N to C terminal direction) on the basis of their occurrence and adjacency in the set of proteins indicated in the right panel. Mousing over a protein in the panel results in a color change in the network, highlighting the domain architecture of the selected protein as well as revealing the specific orthologs of that protein (useful for a mixed pool of proteins). In this way, the domain architectures in a set of functionally related proteins and their orthologs are easily compared. Further, the network of architectures may be dynamically expanded to include neighboring domains. By selecting a species on the right panel, followed by a node in the graph representing a domain, PhyloPro will retrieve additional domain neighbors corresponding to adjacent domains in all other proteins in that species. These temporary additions are highlighted in a different color reflecting their transient nature. However, a neighbor may be permanently added to the network by right clicking it. Once added to the network in this way the new domain may be used to seed further exploration. Finally, a path of nodes representing an *ad hoc* architecture can be highlighted by double clicking a series of nodes. Each time a node is double clicked it is assigned a number representing its order in this search architecture. Note it is possible to select a node repeatedly. Clicking on the ‘Protein Search’ button will activate a search against the reference species for any proteins containing that pattern of domains. If the resulting proteins contain additional domains, both the additional proteins and their domains will be included in the view. The proteins must contain all three domains in the order specified but the pattern need not be contiguous, i.e. for a search architecture ABC a protein with architecture ADBC will match. It has been shown that domains do not necessarily need to be contiguous in order to contribute to a conserved three-dimensional fold (22, 35, 36). We have further discussed the importance of conserved, higher-order domain architectures elsewhere (23). As the domain adjacency view tool develops, we will seek to respond to user requests to incorporate additional features.

## Conclusions and future plans

A number of caveats are associated with orthology detection (37, 38). First, in the absence of detailed phylogenetic analyses, domain gains, losses and shuffling events can significantly complicate orthology assignments. Second, horizontal gene transfer introduces an additional problem of xenologs which can lead to confounding outcomes. Third, the quality and coverage of genome annotation varies significantly between genome projects. Genomes of lower quality or with lower fold coverage may be associated with incomplete proteomes, giving rise to apparently missing orthologs. Finally, low quality or incomplete gene model annotations due to, for example incorrect splice sites or merging of unrelated genes can result in protein domains being missed and/or erroneous orthology assignments (for a more in depth discussion of the effects of genome annotation errors on the evaluation of domain architectures, see Ref. 17). While attempts have been made to define the quality of genomes based on metrics such as presence of indels (39) or expectations of gene content (40), we note that there has been no systematic evaluation of genome quality. Further, the choice of genome inclusion is also dependent on the additional value that a genome brings to an analysis (e.g. increasing phylogenetic coverage). Consequently, we chose to use published genomes that provide a good compromise between phylogenetic coverage and status of genome assembly. Reliance on the use of Pfam-defined domains, while subject to biases in the choice of organisms to generate seed alignments for the definition of domains, nonetheless provides a well-established framework to study domain evolution. However, while future versions of PhyloPro will explore the integration of additional sources of domain predictions, the user should be aware that the current reliance on Pfam definitions may result in errors, such as missed domains, in some descriptions of domain architectures.

Given the recognition of the need for standards (18, 41), we anticipate that future updates of PhyloPro will exploit more comprehensive sources of ‘standardized’ genome assemblies that provide comparable accuracies and coverage. Efforts by the ‘Quest for Orthologs’ consortium (http://questfororthologs.org) have resulted in some progress (42, 43). For example, the development of xml-based file exchanges formats (SeqXML, OrthoXML) as well as benchmarks for algorithm comparison. Nevertheless, a range of methods for determining orthology exist and will likely continue to exist given different approaches for optimizing computational efficiency, scalability or for focusing on specific phylogenetic groups differing in characteristics (e.g. homogeneity/diversity, introns, multidomains) (10, 18). The availability of large sets of complementary ortholog predictions from tree-based approaches, e.g. PhylomeDB (9) or integrated in the form of MetaPhOrs (10) highlights directions for future expansion of PhyloPro to include alternative sources of ortholog prediction as a way of increasing overall accuracy of assignments. Similarly, complementary sources for domain predictions exist, e.g. the NCBI’s Conserved Domain Database (44), SMART (45) and InterPro (46) and represent an opportunity for the incorporation of additional tracks. At the same time, integration of domain architectures into orthology prediction pipelines may offer an additional route to help resolve complex orthology relationships. Such approaches have recently been applied to decrease search space associated with exhaustive sequence comparisons (47), but have also shown promise in improving homolog assignments (48). Given the InParanoid pipeline allows the definition of one–many and many–many orthologous relationships, it may be possible in future studies, to infer through interrogation of domain architectures which, among the set of inparalogs presented, represents the true ortholog.

Recent discussions have highlighted the potential importance of sequence and domain-based similarity approaches for the inference of functional similarity compared with tree-based phylogenetic approaches that appear to more closely adhere to the original definition of orthology as a pattern of inheritance (14, 18). PhyloPro is an innovative sequence-similarity-based resource to incorporate domain-level information together with significant tools enabling the exploratory analysis of domain conservation across species. Applied to pathways or complexes, PhyloPro facilitates the rapid identification of core conserved elements of biological processes and potential lineage-specific innovations.

## Funding

Canadian Institute of Health Research (MOP# 84556); the Natural Sciences and Engineering Research Council of Canada (RGPIN-2014-06664). N.L. received a postdoctoral fellowship, in part, through the Hospital for Sick Children Research Training Centre. High performance computing resources were provided by the SciNet HPC Consortium at the University of Toronto.

*Conflict of interest*. None declared.
